# Unveiling the Role of Nano-Formulated Red Algae Extract in Cancer Management

**DOI:** 10.3390/molecules29092077

**Published:** 2024-04-30

**Authors:** Gopalarethinam Janani, Agnishwar Girigoswami, Balasubramanian Deepika, Saranya Udayakumar, Koyeli Girigoswami

**Affiliations:** Medical Bionanotechnology, Faculty of Allied Health Sciences, Chettinad Hospital and Research Institute, Chettinad Academy of Research and Education, Chettinad Health City, Kelambakkam, Chennai 603103, India; jananigopalarethinam98@gmail.com (G.J.); deepikabalu70@gmail.com (B.D.); usaranyaudayakumar@gmail.com (S.U.)

**Keywords:** *Amphiroa anceps*, nanoformulation, anticancer activity, cell viability, zebrafish embryos

## Abstract

Cancer is one of the major causes of death, and its negative impact continues to rise globally. Chemotherapy, which is the most common therapy, has several limitations due to its tremendous side effects. Therefore, developing an alternate therapeutic agent with high biocompatibility is indeed needed. The anti-oxidative effects and bioactivities of several different crude extracts of marine algae have been evaluated both in vitro and in vivo. In the present study, we synthesized the aqueous extract (HA) from the marine algae *Amphiroa anceps*, and then, a liposome was formulated for that extract (NHA). The extracts were characterized using different photophysical tools like dynamic light scattering, UV–visible spectroscopy, FTIR, scanning electron microscopy, and GC-MS analysis. The SEM image revealed a size range of 112–185 nm for NHA and the GC-MS results showed the presence of octadecanoic acid and n-Hexadecanoic acid in the majority. The anticancer activity was studied using A549 cells, and the NHA inhibited the cancer cells dose-dependently, with the highest killing of 92% at 100 μg/mL. The in vivo studies in the zebrafish model showed that neither the HA nor NHA of *Amphiroa anceps* showed any teratogenic effect. The outcome of our study showed that NHA can be a potential drug candidate for inhibiting cancer with good biocompatibility up to a dose of 100 μg/mL.

## 1. Introduction

Cancer is one of the major global health problems. The survival rate of cancer patients can be increased if they are diagnosed and treated at a very early stage [1]. Even though numerous research studies and developments are carried out in the field of cancer, it remains a killer disease [2]. In India, 1 out of 9 people are expected to develop cancer in their lifetime. Breast and lung cancer top the list [3]. Cancer treatment involves chemotherapy, surgery, and irradiation. Out of all these, chemotherapy is the major conventional therapeutic approach for cancer. However, most chemotherapeutic drugs do not have specificity or solubility, which leads to multidrug resistance [4].

Marine seaweeds are rich in vitamins, lipids, amino acids, proteins, etc. Studies on these bioactive compounds have revealed that they have anti-inflammatory, antimicrobial, antioxidant and anticancer activities [5]. Seaweed can be divided into three classes: red (Rhodophyta), brown (Phaeophyta), and green (Chlorophyta) algae. Out of these, red algae possess many bioactive metabolites and are known for their anticancer and antiviral activities. They also have an additional pigment called phycoerythrin, which gives them their red color [6,7]. The red alga *Amphiroa anceps* is a universal seaweed inhabiting temperate to tropic regions. It contains several bioactive compounds like gallic acid, ellagic acid and phenolic compounds. The bioactive components of this red seaweed have been studied previously [8]. Karthick et al., in 2019, studied the antibacterial activity of the benzene, acetone, and ethanolic extracts of *Amphiroa anceps*. They found that the ethanolic extract showed the maximum zone of inhibition against *Vibrio parahaemolyticus*, and the benzene extract showed maximum inhibition against *E. coli* [7]. In another study by Raj in 2016, benzene, methanol, aqueous, petroleum ether, chloroform, acetone, and isopropanol extractions of *Amphiroa anceps* were prepared. The phytochemical and HPLC analysis revealed the presence of sugars, phenolic compounds, tannins, saponins, alkaloids, and glycosides [9]. Tanna et al., in 2022, studied the methanolic extract of *Amphiroa anceps* for its antioxidant, radical scavenging, and anticancer activity. *Amphiroa anceps* showed potential antioxidant and anti-proliferation activity, proving it to be a potential candidate for nutraceutical applications [10]. 

Evidence exists that resistance develops to drugs and radiation that are repeatedly used to kill cancer cells [11]. To overcome these limitations of conventional cancer treatment strategies, nanomedicines have recently been employed in cancer treatment. Nanotherapeutics overcome the limitations and reduce side effects by their high specificity, biocompatibility, enhanced permeability retention, and increased host immunological response [12]. There are several nanodrug delivery systems available [13,14]. This delivery system (i) can incorporate both hydrophobic and hydrophilic drugs, (ii) can be administered through any route like nasal, ocular, oral, etc., (iii) increases the shelf life of drugs, and (iv) improves drug bioavailability [15]. Currently, a variety of nanocarriers, such as liposomes, polymeric nanoparticles, micelles, nanotubes, etc., are already on the market or under research and evaluation for cancer treatment [16]. Liposomes are the most investigated nanocarriers for delivering the drugs. More than 500 liposomal formulations are in different phases of clinical trials. Due to their pharmacokinetic and pharmacodynamic properties, they are involved in treating various diseases from cancer to inflammatory diseases [17]. The liposome comprises an aqueous compartment surrounded by one or more lipid bilayers [18]. They protect the drug from degradation, reduce drug-related nonspecific toxicity, and can be produced and formulated easily for target-specific delivery [19]. These liposomes protect the encapsulated drug from gastric juice, thus enhancing the absorption of drugs in the intestine [20].

The amalgamation of natural compounds from marine sources and nanotechnology has been explored in this study for superior cancer management. So far, many applications of the red seaweed *Amphiroa anceps* have been studied, but there has been no exploration of the liposomal formulation of the aqueous extract of the dried seaweed. In the present study, the liposomal formulation was performed using phospholipids isolated from a hen’s egg yolk. The liposome-formulated extract was characterized using various photophysical tools. The anticancer activity of the aqueous extract (HA) and liposome formulated aqueous extract (NHA) was studied in A549 (lung cancer cell line). The effect on normal cells was also studied using Chinese hamster lung fibroblasts (V79 cell line). The in vivo toxicity study was carried out in zebrafish embryos. From our study, we could conclude that NHA effectively and specifically inhibited the cancer cells and did not elicit any teratogenic effect. Further, it was demonstrated that the NHA killed the cancer cells through the apo-necrosis pathway.

## 2. Results and Discussion

### 2.1. Phytochemical Analysis of the Aqueous Extract of Amphiroa anceps

The phytochemical analysis showed that the aqueous extract of *Amphiroa anceps* contained tannins, saponin, flavonoids, alkaloids, dextrin (polysaccharide), and carbohydrates. These compositions may contribute towards the inhibition of cells that are undergoing uncontrolled cell growth. Figure S1 and Table S1 show the different phytochemicals present in the aqueous extract (HA) of *Amphiroa anceps*.

### 2.2. Characterization of the Aqueous (HA) and Liposome-Formulated Aqueous Extract (NHA) of Amphiroa anceps

#### 2.2.1. FTIR Analysis

**Figure 1:**
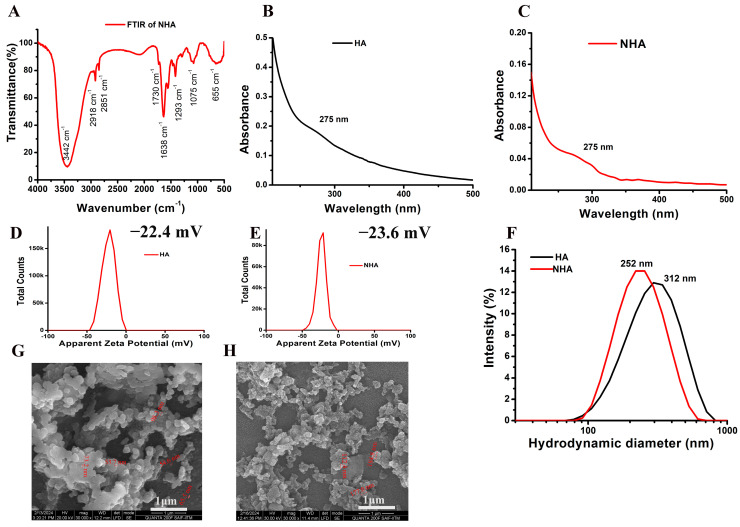
(**A**) The FTIR spectra of NHA of *Amphiroa anceps*. (**B**) The absorption spectrum of the synthesized HA. (**C**) The absorption spectrum of the NHA. (**D**) The zeta potential of HA. (**E**) The zeta potential of NHA. (**F**) The hydrodynamic diameter measures the dynamic light scattering of the HA and NHA. (**G**) Illustrates SEM image of HA. (**H**) Illustrates SEM image of NHA.

The characteristic bonds present in the sample were analyzed using FTIR spectroscopy. The compounds and molecular groups of organic compounds that possess characteristic vibrational, stretching, and bending frequencies in the range of infrared (IR) can be identified using FTIR [21]. The FTIR spectra for the visible peaks are present at 3442 cm^−1^, 2918 cm^−1^, 2851 cm^−1^, 2088 cm^−1^, 1730 cm^−1^, 1638 cm^−1^, 1075 cm^−1^ and 655 cm^−1^ for NHA, as shown in Figure 1A. This represents the presence of O–H stretch and H-bonded peaks, C–H stretch, C=O stretch, N–H bend, C–N stretch and C=C–H; C–H bend. The resultant bands from the FTIR spectra showed similar peaks to the already reported data [22].

#### 2.2.2. Absorption, Size, and Zeta Potential

The absorption spectrum of HA and NHA is shown in Figure 1B and Figure 1C, respectively. The absorption maximum was obtained at 275 nm for both HA and NHA with a typical shoulder-shaped peak. The stability of a nanoparticle in an aqueous environment was measured using surface charge [23]. The zeta potential of the synthesized HA and NHA was found to be −22.4 mV ± 8.24 mV and −23.6 mV ± 6.64 mV, respectively, as shown in Figure 1D and Figure 1E. The value shows that both HA and NHA are highly stable in the aqueous environment. Dynamic light scattering is also known as quasi-elastic light scattering or photon correlation spectroscopy. The scattered light of a sample is measured in very short periods of time and correlates with the data. When the light passes through a sample, the particles are illuminated, and the intensity of the scattered light fluctuates at a rate dependent upon the particle’s size. Analysis of these intensity fluctuations yields the velocity of the Brownian motion and the particle size using the Strokes–Einstein relationship [24]. The hydrodynamic diameter of the HA and NHA was found to be 312 nm ± 127 nm and 252 nm ± 93 nm, respectively, as shown in Figure 1F. The decrease in the particle size of NHA may be due to the physical force acting between the liposomes for entrapping the extract.

#### 2.2.3. Surface Morphology and Encapsulation Efficiency

The scanning electron microscopic image shows the surface morphology of the synthesized HA and NHA. From Figure 1G, it is seen that the particle size of the HA is in the range of 53–98 nm with spherical morphology, and NHA is in the range of 112–185 nm (Figure 1H). The increase in particle size may be due to the entrapment of the drug inside the liposome. The surface of the NHA is a little irregular compared to only HA, indicating a change in molecular arrangements. The encapsulation efficiency of the synthesized NHA was found to be 65% ± 6%.

#### 2.2.4. GC-MS Analysis of HA

**Figure 2:**
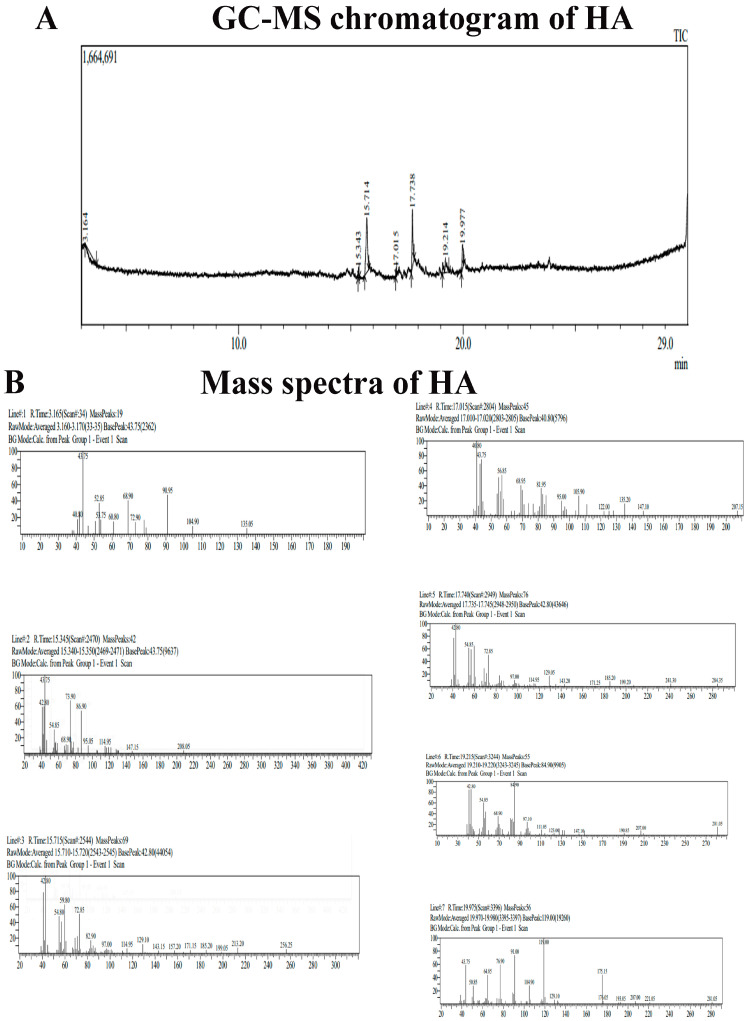
(**A**) The phytochemicals identified are shown as chromatograms. (**B**) Mass spectra data of HA.

Figure 2 showed seven peaks, indicating the presence of seven phytochemical compounds whose mass spectra data are also presented in Figure 2B. In Table 1, the phytochemical constituents with their retention time and concentration (peak area %) are presented. Among the seven compounds identified, the most abundant compounds were n-Hexadecanoic acid with a peak area of 50.36%, and octadecanoic acid with a peak area of 31.64%. These bioactive agents serve as antioxidants and anti-neoplastic agents. Octadecanoic acid has a role in inducing apoptosis.

### 2.3. Cytotoxicity Assay

#### 2.3.1. MTT Assay

**Figure 3:**
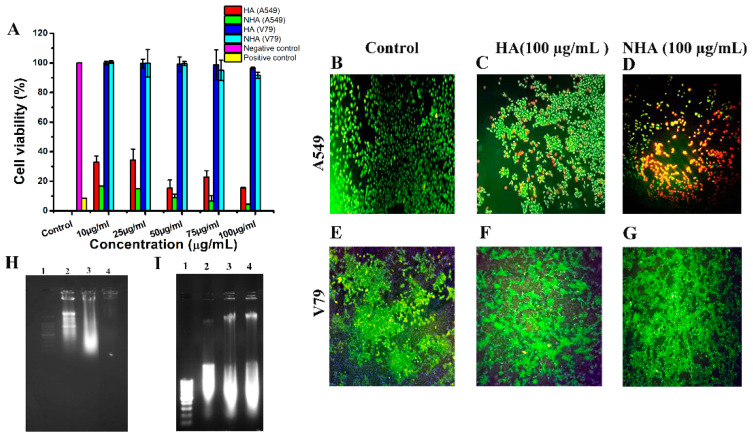
(**A**) MTT assay was carried out in A549, and V79 cells treated with different concentrations of HA and NHA; (**B**–**G**) Percentage of cell death after treatment with 100 μg/mL of HA and NHA in A549 and V79 cells was monitored using fluorescence microscopy. Electrophoresis image of fragmented DNA: lane 1—DNA ladder (100 bp), lane 2—DNA isolated from cells treated with NHA (100 μg/mL), lane 3—DNA isolated from cells treated with HA (100 μg/mL), and lane 4—DNA isolated from untreated cells (**H**) A549 cells and (**I**) V79 cells.

Cell viability assay (MTT) was carried out using cancer cells (A549) and normal fibroblast cells (V79) [25]. The cells were treated with different concentrations of HA and NHA (Figure 3A). The cell viability of A549 cells treated at 100 μg/mL was found to be 16 ± 2% for HA and 4 ± 2% for NHA. From these results, we can conclude that in A549 cells, there was enhanced cell killing after treatment with NHA compared to HA at the same dose. A previous study also reported that the organic extract of *Amphiroa anceps* reduced the cell viability in breast and colon cancer cells [26]. In V79 cells, there was no significant killing in both aqueous and liposome-formulated treated cells. The cell viability at 100 μg/mL for HA and NHA was found to be 96 ± 3% and 92 ± 1%. These results show that HA and NHA can specifically kill cancer cells and do not alter the survival of normal fibroblast cells. 

#### 2.3.2. Live–Dead Assay

To visualize the cytotoxicity of drugs, a live–dead assay was carried out using the dyes ethidium bromide (EtBr) and acridine orange (AO) [27]. Figure 3B–G shows live–dead cell assay results of A549 cells. The control group (Figure 2B) showed green cells, a few red cells (Figure 3C) in the HA-treated group, and many red cells in the NHA-treated groups (Figure 3D). The percentage of cell death was found to be 26 ± 4% for cells treated with HA and 64 ± 3% for NHA-treated cells. Figure 3E–G shows the effect of HA and NHA treatment in V79 cells. The fluorescent microscope images showed no cell killing in fibroblasts after HA and NHA treatment. Nanomedicine is known for its target specific action [28], and our results show that the formulated drug is more specific to cancer cells. 

#### 2.3.3. Morphological Change Observation

The typical inverted images of A549 cells treated with NHA showed morphological changes, such as bulging and shrinkage of cells in Figure S2(2-3), which is a primary indication of necrosis and apoptosis. The control cells and cells treated with HA in Figure S2(2-1,2-2) showed normal morphology. There was no morphological change in normal cells (V79) in both HA- (Figure S2(2-5)) and NHA- (Figure S2(2-6)) treated cells. The cell morphology was similar to that of the control cells (Figure S2(2-4)).

### 2.4. Isolation of Apoptotic/Necrotic DNA

After the isolation of fragmented DNA from the untreated control and HA- and NHA-treated cells (A549 and V79), they were electrophoresed and observed under UV light in a Gel doc (GeNei). After intercalating between the DNA double strand, ethidium bromide emitted fluorescence, which provided evidence of DNA presence. Figure 3H shows the fragmented DNA pattern of A549 cells. Lane (2) from the isolated DNA that was treated with 100 μg/mL of NHA showed both smear and ladder, indicating that the fragmented DNA were contributed from cell death mediated by both apoptosis and necrosis (apo-necrosis). In cells treated with 100 μg/mL of HA, lane (3) showed smear indicating necrosis as the mode of cell death. On the other hand, in lane (4), the untreated control exhibited no fragmentation, indicating no or minimum death in the untreated cells. In V79 cells (Figure 3I), the lanes with the untreated control, HA, and NHA treatment exhibited a smear of DNA with the same intensity for all the groups. This DNA smear may have been present due to the background necrosis of the cells. Since the intensity was similar in all the three groups, it can be concluded that the background death was not due to the HA or NHA treatment; it may have been due to natural death. This shows that neither of the extracts has a cytotoxicity effect on the normal cells, which is corroborated by the results of the MTT assay and live–dead assay.

### 2.5. In Vivo Toxicity Study

**Figure 4:**
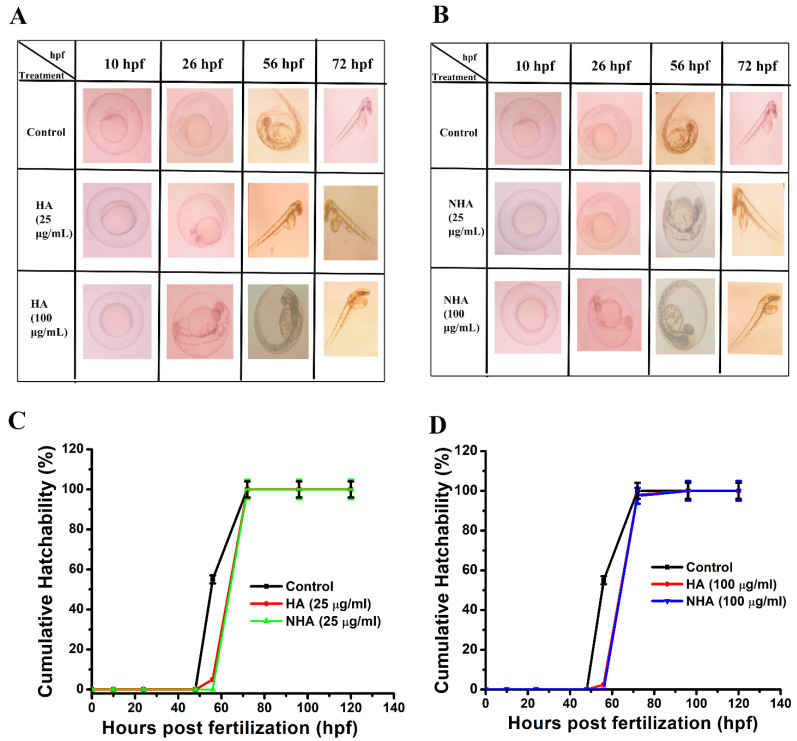
(**A**,**B**) Zebrafish embryo developmental stages at different hpf for HA and NHA was monitored using light microscope. (**C**,**D**) Percent cumulative hatchability of embryos treated with HA and NHA treated at 25 μg/mL and 100 μg/mL.

The teratogenicity effect on the first-week development of zebrafish embryos was monitored under an inverted microscope at regular time intervals (10 hpf, 24 hpf, 48 hpf, 72 hpf, 96 hpf, 124 hpf). There was no morphological change observed even in the high concentration (100 μg/mL) of HA and NHA (Figure 4A and Figure 4B, respectively). The cumulative hatchability percentage of the embryos was also observed (Figure 4C and Figure 4D, respectively), and the percentage of hatchability was 100 ± 2% and 98 ± 4% for the concentrations 25 μg/mL and 100 μg/mL of the HA-treated group, respectively. Whereas for the NHA-treated group, the cumulative percentage of hatchability was 100 ± 2% and 97 ± 3% for the concentrations 25 μg/mL and 100 μg/mL. No delayed hatching was observed in the treatment groups, and the findings were similar to those of the control group. Nanomedicines have the property of enhancing biocompatibility, and they are target specific [29]. These results show that both HA and NHA have no teratogenic effect and are biocompatible up to a dose of 100 μg/mL.

Cancer is considered the uncontrolled proliferation of cells that destroy the surrounding tissue. The resulting aberration results in tumorigenesis and leads to treatment resistance [30]. The number of cancer cases and mortality rates are increasing globally. A few years back, there were only a few treatment options available. But now, many advances have been made in cancer treatment [31,32]. One such advancement is nanomedicine’s involvement in cancer therapy. Nanomedicine uses the tumor environment to bring about target-specific action and exhibit good pharmacokinetic and pharmacodynamic properties [33]. Out of the many nanocarriers available, liposomes naturally have the ability to attack cancer. Many studies revealed that liposomal-formulated drugs are barely toxic compared to nano-encapsulated drugs [34]. Therefore, in our study, the liposomal formulation of marine red alga *Amphiroa anceps* is carried out. Marine algae are exposed to various abiotic and biotic stress. This leads to the production of secondary metabolites that have various bioactive functions [9]. Research and the utilization of marine seaweed have increased worldwide, and pharmacological industries are using it extensively for its pharmacological properties [35]. 

The characterization of the aqueous and liposome-formulated aqueous extracts of *Amphiroa anceps* revealed that they have maximum absorption at 275 nm; the size measured by DLS revealed that after liposome formulation, the size was reduced from 312 nm to 252 nm. It is known that nanoparticles have a reduced surface-to-volume ratio, so their size is small, and this size is the advantage for target-specific action [36]. From the DLS result, we can say that the reduction in size makes the compound work effectively against the cancer cells when compared to only the extract. The formulated compound was stable in its aqueous environment, and the SEM analysis revealed that the size increased from 95 nm to 185 nm. This may be due to the encapsulation of the drug inside the liposomes. The GC-MS analysis revealed that the aqueous extract is rich in bioactive components that are responsible for its anticancer activity. In vitro cytotoxicity analysis revealed that the liposome-formulated extract was more effective in killing the cancer cells compared to only the extract. The aqueous extract kills cancer cells via the necrosis pathway, and the liposome-formulated extract follows both apoptosis and necrosis pathways. The in vivo toxicity study on zebrafish embryos revealed that they do not have any teratogenicity effect in the first-week development stage of the embryos. These findings reveal that liposomal formulation enhanced the anticancer properties of *Amphiroa anceps*. Future studies are needed to monitor the anticancer activities in animal models.

## 3. Material and Methods

### 3.1. Materials

A549 adenocarcinoma human alveolar basal epithelial cells and V79 Chinese hamster lung fibroblasts were purchased from the National Centre for Cell Science (NCCS), Pune. Dulbecco’s Modified Eagle’s Medium (DMEM) and antibiotic solution were purchased from HiMedia, Mumbai, India. Fetal bovine serum (FBS) was purchased from Gibco, Waltham, MA, USA. Other chemicals such as MTT [3-(4,5-dimethylthiazol-2-yl)-2,5-diphenyltetrazolium bromide, acridine orange, ethidium bromide, and cholesterol were purchased from HiMedia, India. The 100 bp DNA ladder and DNA gel-loading dye (6x) were purchased from Thermo Fisher Scientific, Mumbai, India. Dimethyl sulfoxide (DMSO), chloroform, isopropanol, and ethanol were purchased from SRL chemicals, Mumbai, India.

### 3.2. Amphiroa Anceps Extract Preparation

The fresh seaweed had been collected from the Mandapam coast, Ramanathapuram. The seaweed was ground to a fine powder using Mortar and Pestle. The aqueous extract (HA) was prepared by combining and modifying the protocols of Lopez et al., 2010; Haq et al., 2019; and Kumar et al., 2019 [37,38,39]. Around 1 g of the powder was dissolved in 15 mL of distilled water and kept in a magnetic stirrer for 2 h at room temperature. The mixture was then subjected to sonication for 15 min in a bath sonicator (40 KHz) at 40–45 °C, and the extract was centrifuged at 4000 rpm for 20 min. The supernatant was collected in a separate tube, and the pellet was again resuspended in 15 mL of distilled water and kept in a magnetic stirrer for 2 h at room temperature, then sonicated for 15 min in a water bath sonicator (40 KHz) at 40–45 °C, and centrifuged at 4000 rpm for 20 min; finally, the supernatant was collected. Both supernatants were pooled together and filtered using a Whatman filter of pore size 0.45 μm, and the filtrate was dried completely using a vacuum evaporator and dried in a desiccator overnight at room temperature (25–27 °C). 

The yield of the aqueous extract obtained can be calculated using the following formula:

$$Extraction\;yield\;(\%)=\frac{Dry\;weight\;of\;extract\;(g)}{Dry\;weight\;of\;algal\;biomass\;(g)}\times 100$$

The yield of the HA was found to be 2%. The dried residue was then dissolved in 10 mL of phosphate-buffered saline (PBS) at pH = 7.4, and stored at 4 °C until further use.

### 3.3. Liposomal Formulation of Aqueous Extract of Amphiroa anceps

The liposomes were prepared using phospholipids. Phospholipids were extracted from the hen’s egg yolk [25]. To 1 mL of the egg yolk, 3 mL of 1M NaCl were added and vortexed. To this content, 12 mL of methanol and 6 mL of a chloroform mixture (2:1 ratio) were added, and they were mixed well. Then, 3 mL of chloroform and 1 mL of 1 M NaCl were added and vortexed. This mixture was subjected to centrifugation at 2000 rpm for 5 min. After centrifugation, three layers formed. Phospholipid was present in the bottom organic phase, and it was collected. The collected phospholipid was pale yellow in color and stored at −20 °C for further use. In the round bottom (RB) flask, the following contents were added. In total, 20 μL of phospholipids which had been isolated from the egg yolk were mixed with 4 mg of cholesterol and 4 mL of methanol and 16 mL of chloroform. To this, 200 μL of HA was added, and a rotatory vacuum pump was used to completely dry the mixture. A thin film was obtained inside the flask walls, and it was kept in the desiccator overnight at room temperature (25–27 °C). The next day, the thin film was sonicated with 20 mL of PBS for 15 min at room temperature (27–30 °C), and then stored at 4 °C for further use.

### 3.4. Characterization

For characterization, tools like Shimadzu UV–visible spectrophotometry for measuring the absorption spectrum, Malvern zeta sizer and dynamic light scattering (DLS) for the measurement of surface charge and hydrodynamic diameter, respectively, Bruker Fourier transformed infrared (FTIR) spectroscopy for the measurement of characteristic bonds present, and JEOL JEM 2100 HRTEM Scanning electron microscopy (SEM) to characterize the surface morphology and size were used. Phytochemical analysis was performed using the standard protocol [40].

### 3.5. GC-MS Analysis of Aqueous Extract of Amphiroa anceps

GC-MS analysis was carried out to identify the secondary metabolites present in the HA. The phytochemicals were identified using Agilent Technologies 7890B gas chromatography coupled with 5977B mass spectrometry. In total, 3 μL of the sample were diluted with methanol. The diluted sample was loaded in DB-5ms column 30 m × 250 μm × 0.25 μm. The initial column temperature was at 60 °C, and the temperature was linearly increased from 60 °C to 310 °C with a 3 min hold. The temperature of the injection port was 280 °C. The sample was injected manually using the split mode, with a helium carrier gas flow rate of 1 mL/min. The total elution time of the sample was 29 min. The compounds were identified based on their retention time, fragmentation pattern, and through NIST-based automated mass spectral deconvolution and identification software.

### 3.6. Encapsulation Efficiency

In total, 200 μL of HA were mixed with 20 mL of PBS (pH = 7.4). The absorbance was measured at the λmax of the liposomal-formulated aqueous extract of *Amphiroa anceps* (NHA) (i.e.,) 275 nm. Then, 1 mL of NHA was centrifuged using REMI RM-03 Plus Micro Centrifuge at 10,000 rpm for 20 min at room temperature (25–32 °C). Without disturbing the pellet, the supernatant was collected, and absorbance was measured at 275 nm. The encapsulation efficiency of NHA was calculated using the formula given below [41].

$$EE\%=\frac{Total\;Drug-Unbound\;Drug}{Total\;Drug}\times 100$$

### 3.7. Cytotoxicity Assays

#### 3.7.1. MTT

An MTT (3-(4,5-Dimethylthiazol-2-yl)-2,5-Diphenyltetrazolium Bromide) assay was carried out to check the cytotoxicity of HA and NHA. For this assay, A549 cells (Lung cancer cells) and V79 (Fibroblast cells) with an equal seeding density of nearly 10^4^ cells/well were cultured on 48-well plates that contained a sterile DMEM medium containing 10% of fetal bovine serum (FBS) and 1% of antibiotics, and incubated with 5% CO_2_ at 37 °C for 24 h under humidified atmosphere. After 24 h of incubation, the cells were treated at different concentrations (10 μg/mL, 25 μg/mL, 50 μg/mL, 75 μg/mL, 100 μg/mL) of HA and NHA and further incubated for 24 h. In A549 cells, Doxorubicin (Dox) was used as drug control at the 1 μg/mL concentration. To the incubated cells, MTT was added at a concentration of 5 mg/mL, and again, the cells were incubated in the dark for 4 h. After 4 h of incubation, 500 μL of DMSO (Dimethyl sulfoxide) were added to dissolve the formazan crystals. The optical density was measured at 570 nm. The percentage of viable cells was calculated as per the formula given [42]. The experiments were repeated thrice.

$$\%\;cell\;viability=\frac{\left(O.D.\right)\;at\;570\;nm\;of\;treated\;cells}{\left(O.D.\right)\;at\;570\;nm\;of\;untreated\;control\;cells}\times 100$$

#### 3.7.2. Live Dead Assay

Fluorescence microscopy was used to assess the number of live and dead cells [27]. To perform a live–dead assay, A549 and V79 cells were cultured in a DMEM supplemented with 10% FBS and 1% antibiotics and incubated with 5% CO_2_ at 37 °C for 24 h. The next day, to seed the cells on sterile coverslips, they were sterilized with 70% ethanol; it was allowed to fully dry for a few minutes, and kept inside a 35 mm Petri plate. After seeding 10^4^ cells on the sterile coverslip, they were kept for further incubation for 24 h. The incubated A549 and V79 cells were treated with 100 μg/mL of HA and 100 μg/mL of NHA and incubated further for 24 h at 37 °C with 5% CO_2_. To visualize the cells, the stock concentration of acridine orange dye was prepared at a concentration of 1.2 mM, and ethidium bromide was prepared at a concentration of 1.9 mM. From the stock solution, the working solution (dye mixture) of dye was prepared by dissolving 5 μL of acridine orange and 2 μL of ethidium bromide in 2 mL of sterile PBS. After 24 h of incubation, the medium was removed, and 1 mL of the dye mixture was added and incubated for 3 min at 37 °C; excess dye was washed with PBS and visualized under a fluorescent microscope. Acridine orange stains the live cells, which appear in green, whereas ethidium bromide stains the dead cells, which appear in red. The percentage of dead cells was calculated using the following formula:

$$\%\;of\;dead\;cells=\frac{Total\;number\;of\;dead\;cells}{Total\;number\;of\;cells}\times 100$$

The experiment was repeated thrice. The images of the cells were captured randomly throughout the coverslip, and the number of live and dead cells was counted.

#### 3.7.3. Morphological Change Observation

The morphological changes were observed for A549 and V79 cells using inverted microscopy. The cells were cultured on sterilized coverslips, and kept in DMEM (Dulbecco’s Modified Eagle Medium) supplemented with 10% of fetal bovine serum (FBS) and 1% of antibiotics and incubated with 5% CO_2_ at 37 °C for 24 h till they attained 80% confluency. After they reached confluency, the cells were treated with 100 μg/mL each of HA and NHA for 24 h at 37 °C with 5% CO_2_. After 24 h, morphological changes were observed using an inverted microscope (Olympus CKX53) at 10X magnification. The experiments were repeated thrice.

### 3.8. Isolation of Apoptotic/Necrotic DNA

Post-treatment with HA and NHA, the cancer cells may die due to apoptosis, necrosis, or apo-necrosis. In the case of apoptosis, typical 200 bp fragments and multiples of 200 bp are obtained because the DNA gets cleaved at the linker regions by the caspase activated DNAses (CADs). Thus, when these DNA are electrophoresed, they show a ladder-like appearance. On the other hand, necrosis is identified with DNA fragments of all sizes, and appears as a smear in DNA agarose gel electrophoresis [43]. To identify the mode of cell death, whether it is apoptotic or necrotic, fragmented DNA were isolated from A549 and V79 cells treated with HA (100 μg/mL) and NHA (100 μg/mL). For DNA isolation, A549 and V79 cells were cultured in equal numbers (5 × 10^5^ cells/plate) in three T25 flasks for each cell type that contained DMEM medium, which was supplemented with 10% of FBS and 1% of antibiotics and incubated with 5% CO_2_ at 37 °C for 24 h. Twenty-four hours after the cell seeding, treatment with HA and NHA was carried out and the cells were further incubated for 24 h. Since the doubling time of A549 and V79 cells is 22–24 h, it was expected that we could observe apoptosis/necrosis 24 h after treatment. Untreated cells were taken as a control, and treated cells were harvested with 0.25% trypsin 24 h after treatment with HA and NHA for fragmented DNA isolation. The harvested cells were centrifuged at 1500 rpm for 5 min, and the cell pellet was washed twice using the sterile PBS (pH-7.4). To the collected pellet, a TKM2 buffer (10 mM Tris HCl, 10 mM KCl, 10 mM MgCl_2_, 2 mM EDTA, 0.4 M NaCl) and 40 μL of 10% SDS were added; it was mixed gently by inverting, and the mixture was further incubated at 55 °C for 5 min. Then, 150 μL of 6 M NaCl were added to this mixture, vortexed, and centrifuged at 12,000 rpm for 10 min at room temperature. Around 600 μL of the supernatant were collected, and to this, around 350 μL of isopropanol were added and inverted slowly to precipitate the DNA. The mixture was again centrifuged at 12,000 rpm for 5 min. To the pellet, 500 μL of 70% ethanol were added and centrifuged at 10,000 rpm for 5 min and this ethanol wash was repeated once more. The pellet was then kept for air drying. After the complete evaporation of ethanol, the pellet was dissolved in 10–30 μL of a TE buffer (10 mM Tris HCl and 1 mM EDTA) and stored at −20 °C. A 1% agarose gel, which contained 0.5 μg/mL of ethidium bromide, was used to run the DNA. The isolated DNA was observed, and the image was captured using a UV illuminator (GeNeiTM) [43]. The experiment was repeated thrice.

### 3.9. Cumulative Hatchability Assay

Zebrafish embryos were used to study the in vivo toxicity of the HA and NHA. The necessary ethical clearance was obtained from the Institutional Animal Ethics Committee (IAEC)—reg no: IAEC-1/Proposal:148/A.Lr.:111/Dt:20.02.2024. The embryos were treated with different concentrations (25 μg/mL and 100 μg/mL) of the drug to study their teratogenicity effect in the first week of development. In a 6-well plate, 75 eggs (5 eggs/well) were divided into three groups; namely, the control group, which was provided with only an E3 medium, HA group, and NHA group. All three groups were maintained in the same condition. The medium in all the wells was changed daily. At regular time intervals of hours post-fertilization (hpf) (10 hpf, 24 hpf, 48 hpf, 72 hpf, 96 hpf, 124 hpf), the embryos were observed under the inverted microscope (Olympus CKX53) and the images were captured. The method described by Harini et al., 2024 [44], was adopted to calculate the cumulative hatchability percentage. The experiment was repeated thrice as follows:

$$Cumulative\;hatchability\;\%=\frac{Nh}{Ni}\times 100$$

where Nh is the number of eggs hatched, and Ni is the initial number of eggs taken. 

## 4. Statistical Analysis

All the experiments were performed thrice. In vitro and in vivo analysis data are represented as mean ± standard deviation. To find the statistical significance level, Student’s *t*-test was used. 

## 5. Conclusions

Cancer occurs due to many unknown factors, and its complex nature makes it resistant to various traditional treatments. To overcome this difficulty, liposomes can be used, which escape the intestinal barrier due to their small size and cell membrane-like nature. Our study elucidated the anticancer activity of the liposomal-formulated aqueous extract (NHA) of Amphiroa anceps. The synthesized aqueous extract (HA) was well encapsulated in liposomes, having an encapsulation efficiency of 65%, and they were stable, having the zeta potential value of −23.6 mV. GC-MS analysis revealed phytochemical components that have a role in anti-neoplastic potential. To confirm the anticancer activity, in vitro analysis was carried out in the A549 lung cancer cell line, and the percentage of killing was found to be 92%. Isolated DNA from NHA-treated cells showed that they triggered the apo-necrosis pathway. Both HA and NHA did not show any in vivo toxicity up to 100 μg/mL. From these results, we can conclude that Amphiroa anceps possess anticancer activity, and it was enhanced by liposomal formulation. The outcome of our study indicated that NHA can be used for the management of cancer after standardizing the dose depending on the type of cancer. Further research needs to be conducted on animal models.

## Figures and Tables

**Table 1:** The major compounds identified by GC-MS and their molecular formula.

S. No.	Name of the Compound	Retention Time	Peak Area%	Molecular Weight	Molecular Formula
1	Propanamide, 3,3,3-trifluoro-2-(trifluoromethyl)-	3.165	21.96	195	C_4_H_3_F_6_NO
2	Decanoic acid, methyl ester	15.345	3.03	186	C_11_H_22_O_2_
3	n-Hexadecanoic acid	15.715	50.36	256	C_16_H_32_O_2_
4	4-Tridecene, (Z)-	17.015	2.48	182	C_13_H_26_
5	Octadecanoic acid	17.740	31.64	284	C_18_H_36_O_2_
6	n-Nonadecanol-1	19.215	17.86	284	C_19_H_40_O
7	2-Pyrazoline-3-carboxylic acid, 5-hydroxy-1-(4-methylbenzoyl)-5-phenyl-, methyl ester	19.975	16.59	338	C_19_H_18_N_2_O_4_

## Data Availability

The data presented in this study are available on request from the corresponding author due to privacy.

## Supplementary Materials

The following supporting information can be downloaded at: https://www.mdpi.com/article/10.3390/molecules29092077/s1. Table S1. The phytochemicals present in aqueous extract of *Amphiroa anceps*. Figure S1: Phytochemical analysis of the aqueous extract of *Amphiroa anceps*. The images show the results of (A)—Foam test; (B)—Baymer’s Test; (C)—Salkowski test; (D)—Ferric chloride test; (E)—Alkaloid test; (F)—Sulphuric acid test; (G)—Glycoside test; (H)—Molisch test; (I)—protein test; (J)—Iodine test. Figure S2: The inverted microscopic image of the A549 and V79 cells treated with aqueous extract and liposome formulated aqueous extract of *Amphiroa anceps* at a dose of 100 μg/ml for 24h. (2-1–2-3) shows control and treated cells with HA and NHA for A549 cells; (2-4–2-6) shows control and treated cells with HA and NHA for V79 cells respectively.
