# Supplementary material for: Unveiling the Role of Nano-Formulated Red Algae Extract in Cancer Management
Source: Molecules. 2024 Apr 30;29(9):2077. doi: 10.3390/molecules29092077 (PMC11085645; doi:10.3390/molecules29092077)
Supplement: Supplementary file 1 [file molecules-29-02077-s001.zip › molecules-2951955-supplementary.pdf]

Supplementary Materials

Unveiling the Role of Nano-Formulated Red Algae Extract in Cancer Management

Gopalarethinam Janani, Agnishwar Girigoswami, Balasubramanian Deepika, Saranya Udayakumar and Koyeli Girigoswami *

Medical Bionanotechnology, Faculty of Allied Health Sciences, Chettinad Hospital and Research Institute, Chettinad Academy of Research and Education, Chettinad Health City, Kelambakkam, Chennai 603103, India; jananigopalarethinam98@gmail.com (G.J.); dragnishwar@care.edu.in (A.G.); deepikabalu70@gmail.com (B.D.); usaranyaudayakumar@gmail.com (S.U.)

* Correspondence: author: koyelig@gmail.com; Tel. +91-9600060358

Table S1. The phytochemicals present in aqueous extract of *Amphiroa anceps*.

S. No	Chemical test	Aqueous extract of *Amphiroa anceps*
	**Test for Saponin**	+++
1	Foam Test	+++
	**Test for Tannins**	+++
2	Baymer's test	+++
	**Test for Terpenoids and Steroids**	-
3	Salkowski test	-
	**Test for Flavonoids**	+++
4	Ferric chloride test	+++
	**Tests for Alkaloids**	+++
6	Dragendorff's Test	+++
	**Test for Quinones**	-
7	Sulphuric Acid Test	-
	**Test for Glycosides:**	-
8	Glycoside test	-
	**Tests for Carbohydrates**	+++
9	Molisch Test	+++
10	Protein Test	-
	**Test for Polysaccharides**	+++
11	Iodine Test	+++

Note: +++ → present components; - → absent.

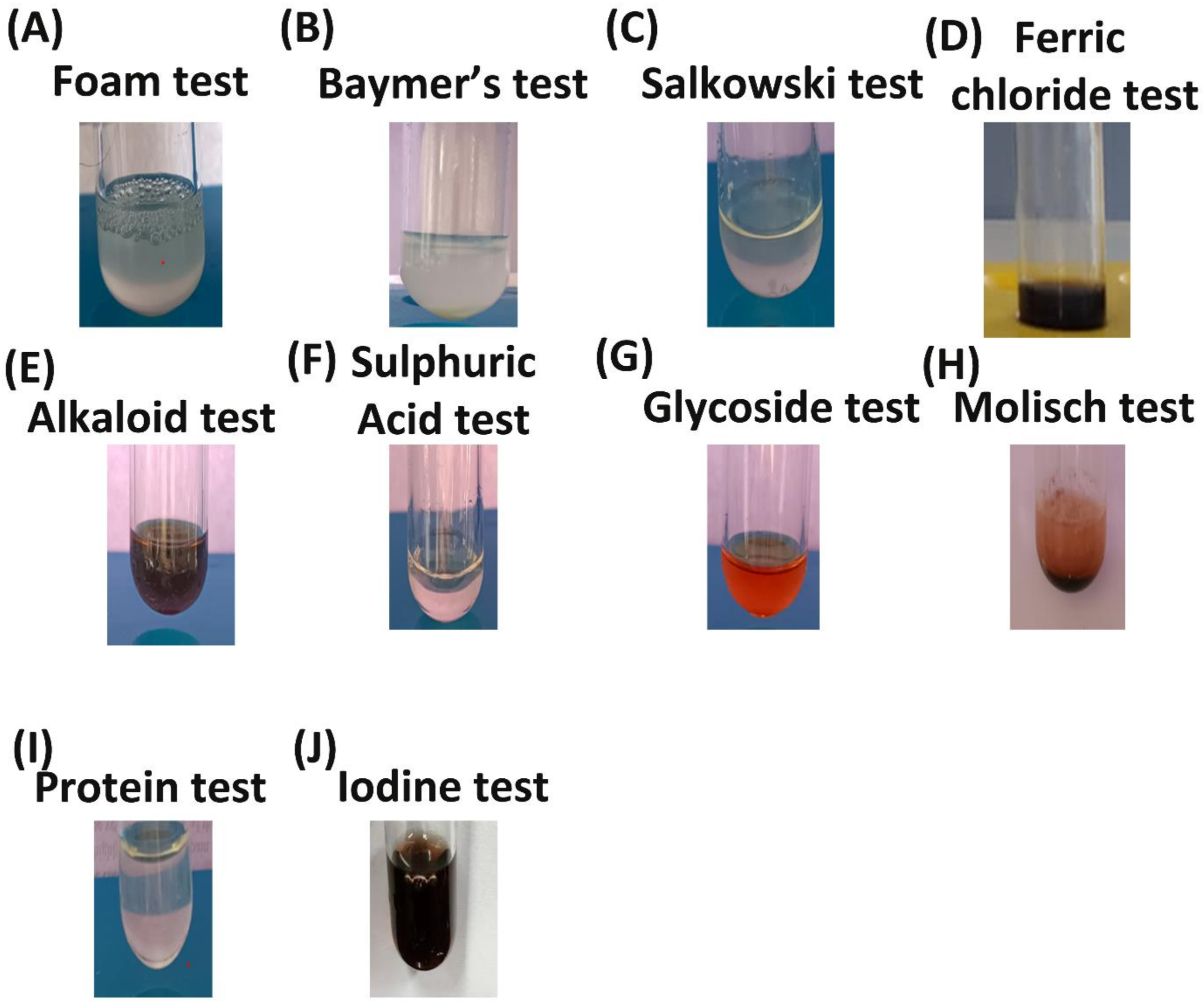

Figure S1. Phytochemical analysis of the aqueous extract of *Amphiroa anceps*. The images show the results of **(A)**-Foam test; **(B)**- Baymer's Test; **(C)**- Salkowski test; **(D)**-Ferric chloride test; **(E)**-Alkaloid test; **(F)**-Sulphuric acid test; **(G)**-Glycoside test; **(H)**- Molisch test; **(I)**- protein test; **(J)**- Iodine test.

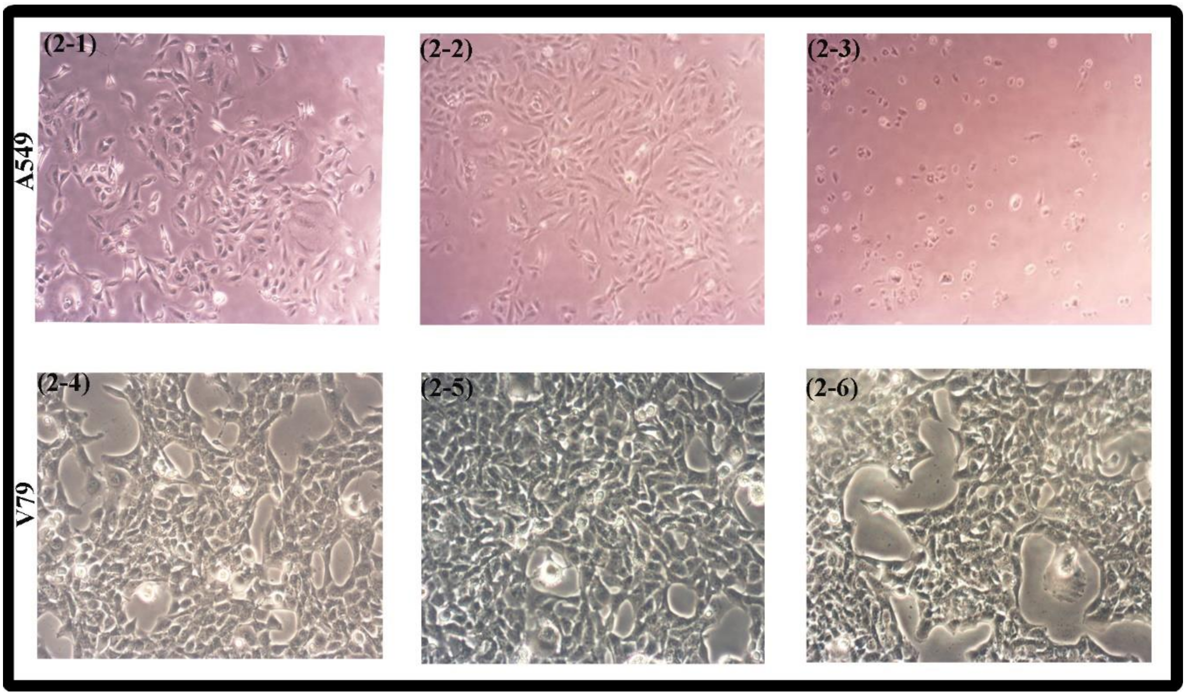

Figure S2. The inverted microscopic image of the A549 and V79 cells treated with aqueous extract and liposome formulated aqueous extract of *Amphiroa anceps* at a dose of 100 µg/ml for 24h. **(2-1; 2-2; 2-3)** shows control and treated cells with HA and NHA for A549 cells; **(2-4; 2-5; 2-6)** shows control and treated cells with HA and NHA for V79 cells respectively.
